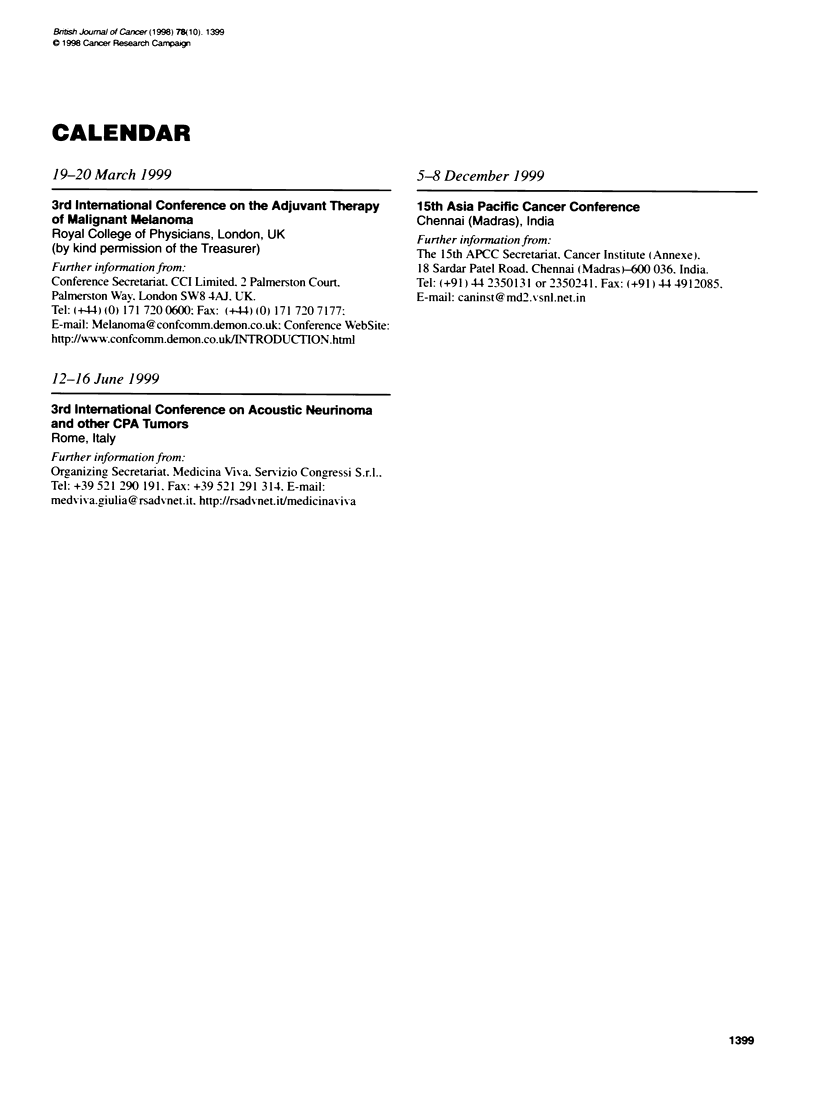# Calendar

**Published:** 1998-11

**Authors:** 


British Journal of Cancer (1998) 78(10). 1399

© 1998 Cancer Research Campaign

### CALENDAR

*19–20 March 1999*

**3rd International Conference on the Adjuvant Therapy of Malignant Melanoma**
Royal College of Physicians, London, UK
(by kind permission of the Treasurer)

*Further information from:*
Conference Secretariat. CCI Limited. 2 Palmerston Court. Palmerston Way. London SW8 4AJ. UK.
Tel: (+44) (0) 171 720 0600: Fax: (+44) (0) 171 720 7177:
E-mail: Melanoma@confcomm.demon.co.uk: Conference WebSite: http://www.confcomm.demon.co.uk/INTRODUCTION.html

*12–16 June 1999*

**3rd International Conference on Acoustic Neurinoma and other CPA Tumors**
Rome, Italy

*Further information from:*
Organizing Secretariat. Medicina Viva. Servizio Congressi S.r.l..
Tel: +39 521 290 191. Fax: +39 521 291 314. E-mail: medviva.giulia@rsadvnet.it. http://rsadvnet.it/medicinaviva

*5–8 December 1999*

**15th Asia Pacific Cancer Conference**
Chennai (Madras), India

*Further information from:*
The 15th APCC Secretariat. Cancer Institute (Annexe).
18 Sardar Patel Road. Chennai (Madras)–600 036. India.
Tel: (+91) 44 2350131 or 2350241. Fax: (+91) 44 4912085.
E-mail: caninst@md2.vsnl.net.in